# Changes in Histone H3 Lysine 36 Methylation in Porcine Oocytes and Preimplantation Embryos

**DOI:** 10.1371/journal.pone.0100205

**Published:** 2014-06-13

**Authors:** Yun Fei Diao, Reza K. Oqani, Xiao Xia Li, Tao Lin, Jung Won Kang, Dong Il Jin

**Affiliations:** Department of Animal Science & Biotechnology, Research Center for Transgenic Cloned Pigs, Chungnam National University, Daejeon, Korea; National University of Singapore, Singapore

## Abstract

Histone H3 lysine 36 (H3K36) methylation is known to be associated with transcriptionally active genes, and is considered a genomic marker of active loci. To investigate the changes in H3K36 methylation in pig, we determined the mono-, di-, and tri-methylations of H3K36 (H3K36me1, H3K36me2 and H3K36me3, respectively) in porcine fetal fibroblasts, oocytes and preimplantation embryos by immunocytochemistry using specific antibodies and confocal microscopy. These analyses revealed that only H3K36me3 in porcine fetal fibroblasts consistently colocalized with transcription sites identified as actively synthesizing RNA based on fluorouridine (FU) incorporation. Treatment of cells with flavopiridol, which blocks transcription elongation, completely abrogated both H3K36me3 signals and RNA synthesis. All three types of H3K36 methylation were present and did not significantly differ during oocyte maturation. In parthenogenetic embryos, H3K36me1 and -me2 were detected in 1-cell through blastocyst-stage embryos. In contrast, H3K36me3 was not detected in most 1-cell stage embryos. H3K36me3 signals became detectable in 2-cell stage embryos, peaked at the 4-cell stage, decreased at the 8-cell stage, and then became undetectable at blastocyst stages in both parthenogenetic and in vitro-fertilized (IVF) embryos. Unlike the case in IVF embryos, H3K36me3 could not be demethylated completely during the 1-cell stage in somatic cell nuclear transfer (SCNT) embryos. These results collectively indicate that H3K36me3, but not H3K36me1 or -me2, is associated with transcription elongation in porcine fetal fibroblasts. H3K36me3 is developmentally regulated and may be a histone mark of embryonic gene activation in pig. Aberrant H3K36 tri-methylation occurred during the nuclear reprogramming of SCNT embryos.

## Introduction

During mammalian fertilization, maternal and paternal chromatids are combined to form a fully totipotent embryo. In oocytes, gene expression is maintained in a silent state during maturation [1], [2]. When oocytes are fertilized by sperm, the zygotes undergo reprogramming and genome activation, followed by replacement of maternal transcripts with embryonic transcripts that regulate embryonic development [3], [4], [5].

The timing of genome activation is different among species. Embryonic genome activation (EGA) in mice occurs at the 2-cell stage [6], whereas porcine and bovine embryos initiate genome transcription at the 4-cell and 8-cell stage, respectively [7], [8]. Although the mechanisms regulating EGA are still not clear, changes in chromatin structure in the early embryo may play an important role. Chromatin compaction affects the accessibility of proteins that regulate gene expression, such as transcription factors and RNA polymerases [9], [10]. The major events involved in this process include changes in DNA methylation and histone acetylation or methylation [11], [12]. In particular, methylation of histones at specific residues is an important epigenetic modification, playing an essential role in both activating and repressing transcription during embryonic development, depending on which lysine residues are methylated [13], [14], [15], [16], [17]. For example, histone H3 tri-methylated at lysine 4 (H3K4me3) is known to be associated with gene activation [18], [19], [20], [21], whereas histone H3 di-methylated and tri-methylated at lysine 9 (H3K9me2 and -me3) and histone H3 tri-methylated at lysine 27 (H3K27me3) are associated with gene silencing [22], [23].

Histone H3 methylation at lysine 36 (H3K36) is another important post-translational modification that is associated with transcription elongation. In yeast, H3K36 methylation which is mediated by Set2, is associated with transcribed genes and is usually referred to as an activating histone mark [24]. Di-methylation and tri-methylation of H3K36 (H3K36me2 and -me3) are generally associated with actively transcribed genes, whereas only H3K36me3 is positively correlated with transcription rates [25], [26]. H3K36me3 enrichment in the coding region of transcribed genes is a mark of the actively transcribed chromatin associated with transcription elongation [27]. During transcription elongation, the chromodomain of Eaf3, a subunit of the Rpd3S histone deacetylase complex, recognizes Set2-mediated H3K36 methylation; the resulting complex is then recruited in the wake of the transcribing RNA polymerase II [28], [29]. Accordingly, H3K36 methylation is also a mark for histone deacetylation [30]. Without Set2 or Rpd3S, acetylated histones accumulate on open reading frames (ORFs), which can lead to transcription initiation from cryptic promoters within ORFs [31], [32]. Thus, Set2 regulates the methylation of histone H3K36, suppressing the incorporation of acetylation and thereby decreasing the initiation of spurious cryptic transcription from within ORFs; this pathway can maintain the accuracy of transcription by RNA polymerase II [33].

Although considerable research on H3K36 methylation has been reported in yeast, there is limited information about H3K36 methylation in mammals. To date, H3K36 methylations in porcine oocytes and preimplantation embryos have not been reported. In the current study, we investigated the changes in H3K36 methylation status in porcine oocytes, and parthenogenetic and in vitro-fertilized (IVF) embryos to determine whether this epigenetic modification is related to genome activation. As in somatic cell nuclear transfer (SCNT) embryos, abnormal epigenetic modification is known to be a major reason for the low efficiency; we also investigated the H3K36 methylation status in SCNT embryos to determine whether these embryos have abnormalities in this epigenetic modification compared with IVF embryos.

## Materials and Methods

### Chemicals and Ethics

Unless otherwise noted, chemicals were purchased from Sigma-Aldrich Chemical Company (St. Louis, MO, USA). All animal care and use procedures were approved by the Institutional Animal Care and Use Committee of Chungnam National University.

### Porcine Oocyte Collection and in vitro Maturation

Porcine ovaries were obtained from a local slaughterhouse (NH Livestock Cooperation Association, Nonsan City, Chungnam Province, Korea) where we had acquired permission to use porcine ovaries, and transported to the laboratory within 2 h in phosphate-buffered saline (PBS) solution supplemented with 100 IU/ml penicillin and 50 µg/ml streptomycin at 30 to 35°C. Cumulus-oocyte complexes (COCs) were obtained from follicles (2–6 mm in diameter) using a 10-ml syringe fixed with an 18-gauge needle. The COCs were washed three times in TL-HEPES containing 0.1% (w/v) polyvinyl alcohol (PVA). The oocytes were then cultured in maturation medium (500 µl per well, see below for details) in 4-well plates (Nunc, Roskilde, Denmark) and incubated for 42 to 44 h at 38.5°C in humidified air containing 5% CO_2_. After 22 h of in vitro maturation, the oocytes were washed three times and transferred to 500 µl of maturation medium without hormones for an additional 20 to 22 h of culture. The maturation medium consisted of TCM-199 (M-4530, Sigma) supplemented with 10% (v/v) porcine follicular fluid, 3.05 mM D-glucose, 0.91 mM sodium pyruvate, 0.57 mM L-cysteine, 0.5 µg/ml LH (L-5269, Sigma), 0.5 µg/ml FSH (F-2293, Sigma), 10 ng/ml epidermal growth factor (E-4127, Sigma), 75 µg/ml penicillin, 50 µg/ml streptomycin, and 0.05% (v/v) MEM vitamins (M-6895, Sigma). Following in vitro maturation, the COCs were transferred to 0.3% hyaluronidase in TL-HEPES-PVA and pipetted repeatedly for 2 min to denude the oocytes of cumulus cells.

### Preparation of Porcine Fetal Fibroblasts

In this manuscript, all animal procedures were approved by the Institutional Animal Care and Use Committee of Chungnam National University. The porcine fetal fibroblasts used in this study were isolated from Korean native pig fetuses at day 35 of gestation. The head and internal tissues were removed using fine scissors, and soft tissues such as liver and intestine were discarded. The remaining tissue was cut into small pieces with fine scissors, treated with 0.05% trypsin and 0.5 mM EDTA (15050-065, Gibco), and shaken for 10 min at 38.5°C. The resulting suspension was centrifuged at 500 rpm for 10 min, and the pellet (containing porcine fetal fibroblasts) was washed three times in DMEM. The cells were resuspended in DMEM containing 75 µg/ml penicillin G, 50 µg/ml streptomycin, 5% (v/v) fetal bovine serum (FBS; 16000-044, Gibco) and 5% (v/v) newborn calf serum (NCS; 26010-074, Gibco), and cultured at 38.5°C. Fetal fibroblasts from passage 5 were used for experiments.

### Parthenogenetic Activation

After maturation, cumulus-free oocytes were transferred to activation solution (0.3 M mannitol, 1.0 mM CaCl_2_·H_2_O, 0.1 mM MgCl_2_·6H_2_O, and 0.5 mM HEPES) and activated with one DC pulse of 1.1 kV/cm for 30 µs using a BTX Electro-Cell Manipulator 2001 (BTX, San Diego, CA, USA). Activated embryos were cultured in Porcine Zygote Medium (PZM-3) supplemented with 0.3% bovine serum albumen (BSA) at 38.5°C in humidified air containing 5% CO_2_.

### *In vitro* Fertilization

In vitro fertilization of oocytes were carried out as described previously by Li *et al*. [34] After maturation, cumulus-free oocytes with first polar body extruded were washed three times with fertilization medium, a modified Tris-buffered medium (mTBM) containing of 113 mM NaCl, 3 mM KCl, 7.5 mM CaCl_2_·2H_2_O, 5 mM sodium pyruvate, 11 mM glucose, 20 mM Tris, 1 mM caffeine, 0.57 mM L-cysteine, and 0.1% wt/vol BSA. 10 ml fresh semen was transferred into a 15-ml tube and centrifuged at 700×g for 3 min. The pellet was then resuspended and washed once with 1 ml mTCM-199 medium consisting of TCM-199 medium supplemented with 26.2 mM NaHCO_3_, 3.05 mM glucose, 0.91 mM Na-pyruvate, 2.92 mM, Ca-lactate·5H_2_O, 75 mg/l kanamycin, and 10% (vol/vol) FBS (16000-044, Gibco) by centrifugation at 700×g for 3 min; washed twice in 1 ml mTBM at 700×g for 3 min respectively. After the last wash, the sperm pellet was resuspended in mTBM medium and the sperm concentration was adjusted to 1×10^6^ sperm/ml. Approximately 15 to 20 oocytes were transferred into 60 µl droplets of fertilization medium covered with mineral oil at 38.5°C in 5% CO_2_ air for 30 min, to which 10 µl diluted sperm was then added. Oocytes were coincubated with sperm for 6 h at 38.5°C in 5% CO_2_ air, then removed the attached sperm by washing in PZM-3. Thereafter, 10 to 15 zygotes were cultured in 40 µl of in vitro culture medium PZM-3 supplemented with 0.3% BSA, and maintained in a 5% CO_2_ atmosphere at 38.5°C.

### Nuclear Transfer

Nuclear transfer, fusion, and activation were carried out as previously described by Diao *et al*. [35]. Cumulus-free oocytes in PZM-3 medium containing 7.5 µg/ml cytochalasin B at 38°C were enucleated by aspirating the first polar body and adjacent cytoplasm with a fine glass pipette. A single donor cell was placed in the perivitelline space of the enucleated each oocyte. SCNT embryos were simultaneously fused and activated with two DC pulses of 1.1 kV/cm for 30-µs each pulse using a BTX Electro-Cell Manipulator 2001 in 0.3 M mannitol medium containing 1.0 mM CaCl_2_·H_2_O, 0.1 mM MgCl_2_·6H_2_O, and 0.5 mM HEPES. Then 10 to 15 embryos were cultured in 40 µl of PZM-3 medium supplemented with 0.3% BSA and maintained in a 5% CO_2_ atmosphere at 38.5°C.

### Flavopiridol Treatment and Transcript Labeling

Fetal fibroblasts at passage 5 were seeded onto coverslips in the wells of 6-well dishes containing 3 ml DMEM (supplemented with 10% FBS) per well and grown to 90% confluence for using in experiments. Cells were treated with 200 nM flavopiridol (F3055, Sigma) in DMEM (containing 10% FBS) for 30 min, followed by combined treatment with 200 nM flavopiridol and 2.5 mM 5-fluorouridine (F5130, Sigma) in DMEM (containing 10% FBS) for an additional 30 min. After treatment, the cells were washed briefly in PBS three times and fixed by incubating for 5 min in 2% paraformaldehyde (PFA; 18814, Polysciences, Inc. PA, USA) in PBS. The cells were then transferred to PBS containing 2% PFA and 1% Triton X-100 (X100, Sigma) for 10 min to fix and permeabilize. The cells were further permeabilized in PBS-PVA containing 0.5% Triton X-100 and 100 mM glycine for 40 min, and blocked for 30 min at room temperature in 3% BSA and 0.3% Triton X-100 in PBS. After washing in PBG [(PBS containing 0.5% BSA and 0.1% gelatin from cold water fish skin (G7041, Sigma)] for 10 min, the cells were incubated in PBG containing 0.3% Triton X-100 and primary antibodies (1∶100 dilution) against 5-bromo-deoxyuridine (BrdU; B2531, Sigma), H3K36me1 (mono-methyl K36; ab9048, Abcam, Cambridge, UK), H3K36me2 (di-methyl K36; ab9049, Abcam, Cambridge, UK) or H3K36me3 (tri-methyl K36; 9763, Cell Signaling, MA, USA) at 4°C overnight. Cells were then washed in PBG for 10 min and incubated with fluorescein isothiocyanate (FITC)-conjugated bovine anti-rabbit (1∶100 dilution; sc-2365, Santa Cruz Biotechnology, CA, USA) or Texas Red (TR)-conjugated goat anti-mouse (1∶100 dilution; sc-2781, Santa Cruz Biotechnology, CA, USA) secondary antibodies in the dark at room temperature for 1 h. Finally, cells were washed twice with PBG (10 min each) and slide-mounted and stained using mounting medium containing the DNA-binding fluorescent dye, diamidino-2-phenylindol (DAPI; H-1200, Vector Laboratories, Inc. Burlingame, CA). Fetal fibroblasts were observed and imaged using a Zeiss laser-scanning confocal microscope (LSM5 Live, Carl Zeiss, Germany) equipped with ×63 objectives and running Zeiss LSM Image Browser software (Zeiss LSM5 Live Release ver. 4.2. SP1 Image Browser software, Carl Zeiss, Germany).

### Immunocytochemistry and Quantification Analysis

Oocytes and embryos were washed in PBS-PVA (containing 0.1% PVA) for 10 min and fixed in 2% PFA in PBS-PVA for 5 min, followed by combined fixation and permeabilization in PBS-PVA containing 2% PFA and 1% Triton X-100 for 10 min. Oocytes and embryos were then sequentially permeabilized in PBS-PVA containing 0.5% Triton X-100 and 100 mM glycine (G7126, Sigma) for 40 min and blocked in 3% BSA and 0.3% Triton x-100 in PBS for 30 min at room temperature. After washing in PBG for 10 min, oocytes and embryos were incubated in PBG containing 0.3% Triton X-100 and primary antibodies (1∶100 dilution) against H3K36me1, H3K36me2, or H3K36me3 at 4°C overnight. Oocytes and embryos were then washed in PBG for 10 min and incubated with secondary antibodies (1∶100 dilution) in the dark at room temperature for 1 h. Finally, oocytes and embryos were washed twice with PBG (10 min each) and slide-mounted and stained using DAPI-containing mounting medium. Oocytes and embryos were observed and imaged using a Zeiss laser-scanning confocal microscope equipped with ×63 objectives and running Zeiss LSM Image Browser software. Quantification of global H3K36 mono-, di- and tri-methylation of nuclei or cytoplasmic areas was performed using Image J software (National Institutes of Health, Bethesda, MD, USA). The border around nuclei was manually delineated according to DNA staining. In addition, at least two different cytoplasmic areas were delineated for normalization to background. The average pixel intensity of the nuclear areas were calculated by Image J, then normalized by dividing by the average pixel intensity of the background areas.

### Statistical Analysis

All experiments were replicated at least three times. Statistical analysis was carried out using statistical product and service solutions (SPSS) 17.0 software (Inc. 233 South Wacker Drive, 11th Floor, Chicago). Global H3K36 mono-, di- and tri-methylation were compared by one way analysis of variance (ANOVA). Bars represent least-squares showed the standard error in each group. P-values <0.05 were considered statistically significant.

## Results

### H3K36 Methylation Status and Association with Transcriptional Activity in Porcine Fetal Fibroblasts

**Figure 1:**
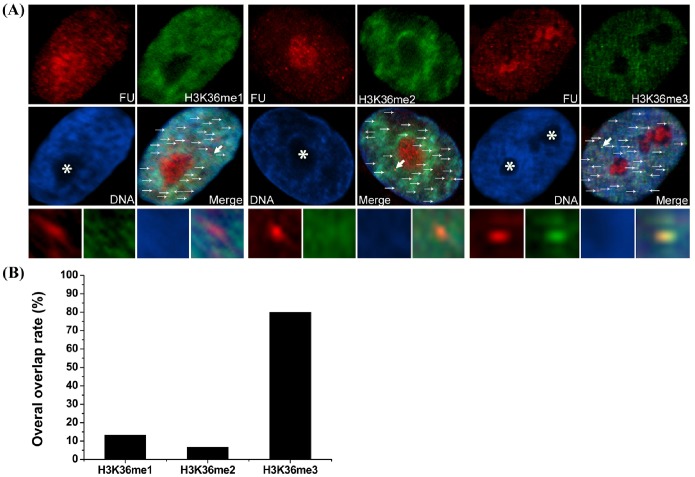
H3K36 methylation status and association with transcriptional activity in porcine fetal fibroblasts. (A) Cells were cultured in medium containing 2.5 mM FU for 30 min, fixed, and immunostained with antibodies against FU and H3K36me1, -me2 or -me3. Primary antibodies were detected with FITC-conjugated (green) and TR-conjugated (red) secondary antibodies. DNA was stained with DAPI (blue). Enlargements (bottom insets) were the area pointed by thick arrows in merge insets showing transcriptional active sites (labeled with FU), H3K36 methylations and their overlap. The asterisk indicates the nucleolus. (B) The overall overlap rates between H3K36 methylations and transcriptional active sites were analyzed using total 30 dots indicated by the thin and thick arrows in (A).

It has been reported that H3K36 methylation is associated with transcription in yeast [25]. Accordingly, to determine whether H3K36 methylation is related to transcription in mammals, we investigated H3K36 methylation status in porcine fetal fibroblasts using H3K36 methylation status-specific antibodies and employed 5-fluorouridine (FU), a cell-permeable, modified RNA precursor, to label active transcription sites. The cells were incubated with 2.5 mM FU in culture medium for 30 min to allow incorporation of FU into nascent RNAs. Cells were then washed briefly with PBS, fixed, and analyzed immunohistochemically using an antibody specific for 5-bromo-deoxyuridine (BrdU) to detect FU labeling, and antibodies against H3K36me1, -me2 or -me3, to detect mono-, di- and tri-methylated H3K36, respectively. As shown in Figure 1, all three types of methylations were observed in interphase cells. H3K36me1 and H3K36me2 labeling was evenly distributed throughout the nucleoplasm, but not in the nucleolus, whereas H3K36me3 exhibited a dotted staining pattern in the nucleoplasm. FU labeling corresponding to nascent transcription site was distributed as dotted patten throughout the nucleus and nucleolus. To analyze the association between H3K36 methylation and nascent RNA synthesis, we assayed one transcriptionally active site (indicated by thick arrow) in each methylation group indicated by a dot where nascent FU-labeled RNA had accumulated. In both H3K36me1 and H3K36me2 groups, transcription sites were not colocalized with methylation sites, indicating that these two types of histone modifications are not marks of transcriptional activity. In contrast, staining for H3K36me3 always exhibited a very clear colocalization with FU-labeled sites, suggesting that this modification may be associated with transcriptional activity. To analyze the overall overlap between FU and H3K36 methylation, we chose 30 dots with strong FU labeling in the nucleoplasm for each methylation group (H3K36me1, -me2 and -me3). We found that 80% of the FU labeling dots were well colocalized with H3K36me3, while only 13.3% and 6.7% of the FU labeling dots overlap with H3K36me1 and -me2, respectively (Fig. 1B).

### Effects of Flavopiridol on H3K36 Methylation and Inactivation of Transcription in Porcine Fetal Fibroblasts

**Figure 2:**
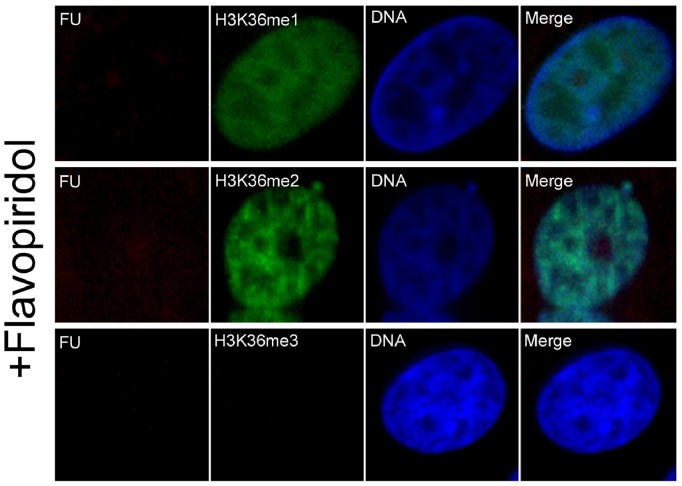
Effect of flavopiridol treatment on H3K36 methylation status in porcine fetal fibroblasts. Cells were treated with 200(FP) for 30 min, followed by combined treatment with 200 nM flavopiridol and 2.5 mM FU for an additional 30 min. Cells were then fixed and immunostained with antibodies against FU and H3K36me1, -me2, or -me3. Primary antibodies were detected with FITC-conjugated (green) and TR-conjugated (red) secondary antibodies. DNA was stained with DAPI (blue).

Flavopiridol is a cyclin-dependent kinase 9 (CDK9) inhibitor, which inhibits transcription by RNA polymerase II in vitro and in vivo by blocking the transition to productive elongation [36], [37]. In preliminary experiments, we confirmed that 200 nM flavopiridol inhibited global transcription more efficiently than 100 nM in porcine fetal fibroblast cells; the transcription could be completely abrogated. Thus, to further investigate the association of H3K36 methylations with transcription, we treated fetal fibroblasts with 200 nM flavopiridol to inhibit transcriptional activity. Cells were treated with flavopiridol and FU and subsequently immunostained with antibodies against BrdU (detecting FU labeling) and H3K36me1, -me2, or -me3. The untreated cells were shown in Figure 1. As shown in Figure 2, after treatment with flavopiridol, transcriptional signals were undetectable. H3K36me3 staining was also absent in these cells, whereas H3K36me1 and -me2 signals still remained intact.

### Changes in H3K36 Methylation Status during Porcine Oocyte Maturation and Preimplantation Development of Parthenogenetic Embryos

**Figure 3:**
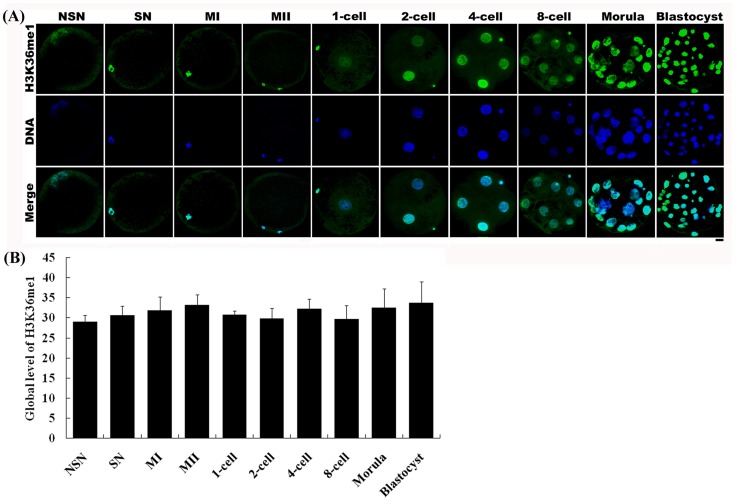
Changes in H3K36me1 status in porcine oocytes and parthenogenetic embryos. (A) Oocytes and parthenogenetic embryos were immunostained with the anti-H3K36me1 antibody, which was then localized with a FITC-conjugated secondary antibody (green). DNA was stained with DAPI (blue). NSN and SN are two types of GV stage oocytes. MI, metaphase I stage oocytes; MII, metaphase II stage oocytes. Scale bar = 20 µm. (B) Relative intensities of fluorescence signals from H3K36me1. Total 13 oocytes and 21 embryos were analyzed in triplicate in this experiment. Bars represent least-squares showed the standard error in each group. P-values <0.05 were considered statistically significant. There was no significant difference among all groups.

**Figure 4:**
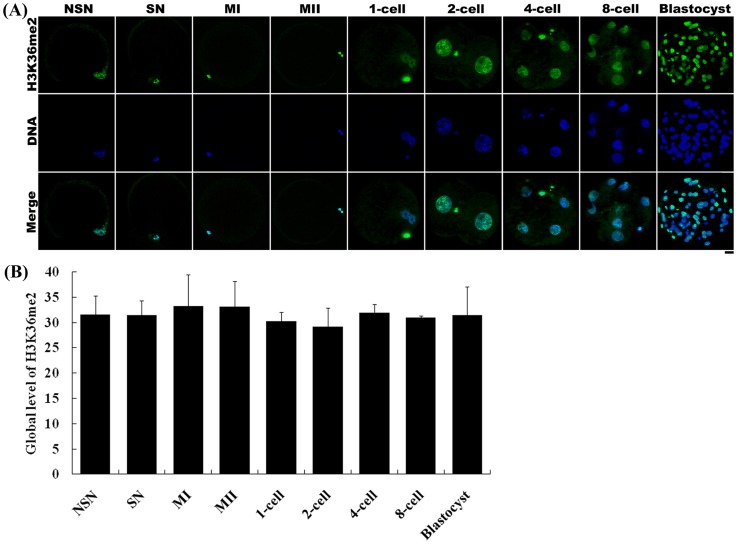
Changes in H3K36me2 status in porcine oocytes and parthenogenetic embryos. (A) Oocytes and parthenogenetic embryos were immunostained with the anti-H3K36me2 antibody, which was then localized with a FITC-conjugated secondary antibody (green). DNA was stained with DAPI (blue). NSN and SN are two types of GV stage oocytes. MI, metaphase I stage oocytes; MII, metaphase II stage oocytes. Scale bar = 20 µm. (B) Relative intensities of fluorescence signals from H3K36me2. Total 15 oocytes and 22 embryos were analyzed in triplicate in this experiment. Bars represent least-squares showed the standard error in each group. P-values <0.05 were considered statistically significant. There was no significant difference among all groups.

**Figure 5:**
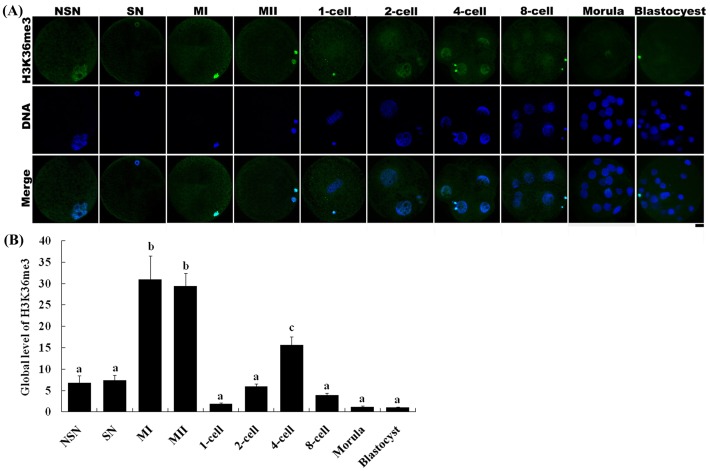
Changes in H3K36me3 status in porcine oocytes and parthenogenetic embryos. (A) Oocytes and parthenogenetic embryos were immunostained with the anti-H3K36me3 antibody, which was then localized with a FITC-conjugated secondary antibody (green). DNA was stained with DAPI (blue). NSN and SN are two types of GV stage oocytes. MI, metaphase I stage oocytes; MII, metaphase II stage oocytes. Scale bar = 20 µm. (B) Relative intensities of fluorescence signals from H3K36me3. Total 23 oocytes and 47 embryos were analyzed in triplicate in this experiment. Bars represent least-squares showed the standard error in each group. P-values <0.05 were considered statistically significant. ^a,b,c^ Values represent least-squares with different superscripts are significantly different (P<0.05).

H3K36 methylation status was examined in different stage oocytes using antibodies directed against mono-, di-, or tri-methylated lysines 36 on histone H3 (H3K36me1, -me2, -me3). All three types of methylation on H3K36 were present in germinal vesicle (GV) oocytes, including those with non-surrounded nucleolus (NSN) and surrounded nucleolus (SN), as well as metaphase I (MI) and metaphase II (MII) stage oocytes. The fluorescence intensities of H3K36me1 and -me2 were similar among all oocyte stages (Figs. 3 and 4). In contrast, the fluorescence intensity of H3K36me3 in NSN and SN oocytes was lower than that in MI and MII oocytes (Fig. 5).

H3K36 methylation status was also examined in parthenogenetic embryos using antibodies against H3K36me1, -me2, or -me3. H3K36me1 and -me2 signals were observed throughout all preimplantation development stages, and showed no change in methylation levels between developmental stages (Figs. 3 and 4). However, H3K36me3 was only present in 2-cell to 8-cell stage embryos (Fig. 5). H3K36me3 fluorescence signals rapidly decreased after activation and were detected again at the 2-cell stage. H3K36me3 signals increased gradually with the development of the embryo until reaching a peak at the 4-cell stage. H3K36me3 signals decreased to a very low level by the time the embryo reached the 8-cell stage, and became undetectable in morula and blastocyst stage embryos.

### H3K36me3 Status in Porcine IVF Embryos

**Figure 6:**
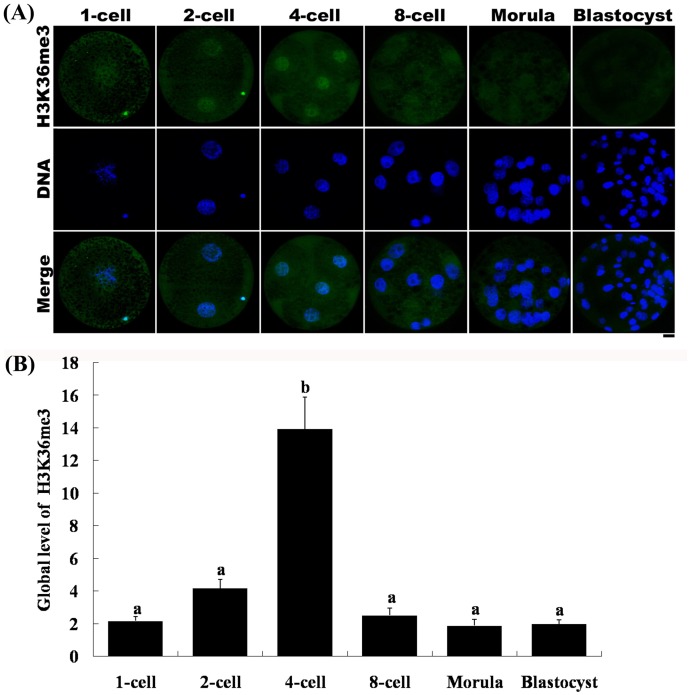
Changes in H3K36me3 status in porcine IVF embryos. (A) IVF embryos were immunostained with the anti-H3K36me3 antibody, which was then localized with a FITC-conjugated secondary antibody (green). DNA was stained with DAPI (blue). Scale bar = 20 µm. (B) Relative intensities of fluorescence signals from H3K36me3. Total 30 embryos were analyzed in triplicate in this experiment. Bars represent least-squares showed the standard error in each group. P-values <0.05 were considered statistically significant. ^a,b^ Values represent least-squares with different superscripts are significantly different (P<0.05).

To identify the dynamic changes in H3K36 tri-methylation in porcine IVF embryos, we immunostained IVF embryos with antibodies against H3K36me3. The results were similar to those obtained with parthenogenetic embryos. Tri-methylation on H3K36 was decreased soon after fertilization, during which the zygotes underwent demethylation (Fig. 6). Similar to the pattern found in parthenogenetic embryos, H3K36me3 signals in IVF embryos were detected at the 2-cell stage and reached their highest level at the 4-cell stage. The intensity of H3K36me3 fluorescence signals was very low, and sometimes undetectable, at the 8-cell stage. By the morula and blastocyst stage, H3K36me3 signals were completely undetectable.

### Aberrant H3K36 Tri-methylation in SCNT Embryos

**Figure 7:**
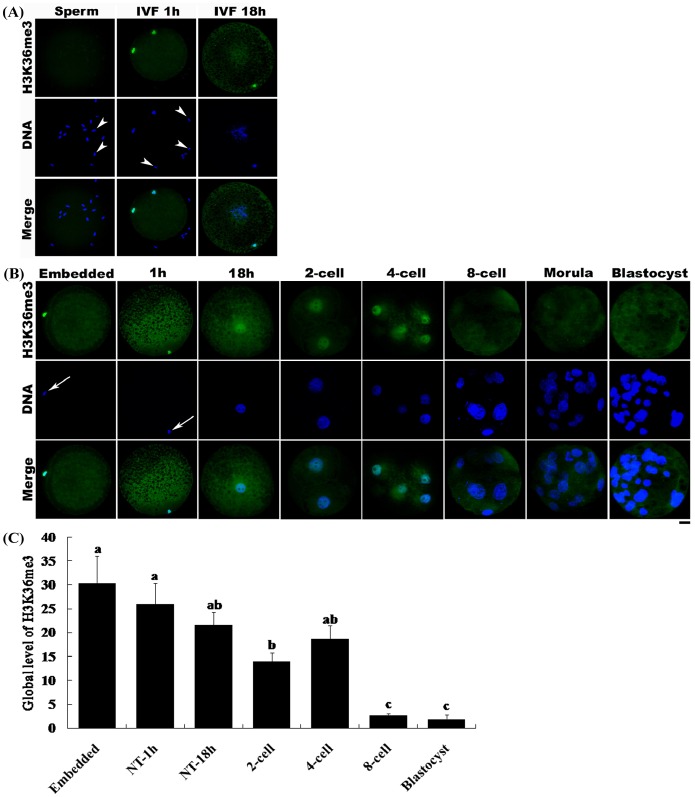
Changes in H3K36me3 status in porcine IVF and SCNT embryos. (A) Sperm, MII stage oocytes coincubated with sperm in mTBM medium for 1 h (IVF 1 h), and IVF embryos cultured in PZM-3 medium for 18 h (IVF) were immunostained with the anti-H3K36me3 antibody. (B) A single fetal fibroblast cell injected into the perivitelline space of a denuded oocyte without activation (Embedded), a nuclear-transferred embryo cultured in PZM-3 for 1 h post activation (1h), a nuclear-transferred embryo cultured in PZM-3 for 18 h after activation (18h), and other stages of SCNT embryos were immunostained with the anti-H3K36me3 antibody. The antibody was localized with a FITC-conjugated secondary antibody (green). DNA was stained with DAPI (blue). The arrowhead indicates sperm and the arrow indicates the nuclei of donor cell. Scale bar = 20 µm. (C) Relative intensities of fluorescence signals from H3K36me3. Total 41 embryos were analyzed in triplicate in this experiment. Bars represent least-squares showed the standard error in each group. P-values <0.05 were considered statistically significant. ^a,b,c^ Values represent least-squares with different superscripts are significantly different (P<0.05).

As shown in Figure 1, H3K36me3 was present in porcine fetal fibroblasts. However, the level of H3K36me3 was low, and even absent, in sperm (Fig. 7A). On the basis of this difference in tri-methylation status between somatic cells and sperm, we investigated whether H3K36 tri-methylation was aberrant in SCNT embryos compared with IVF embryos. Immunostaining of sperm and IVF and SCNT embryos with an antibody against H3K36me3 revealed a very weak or absent H3K36me3 fluorescence signal in sperm (Fig. 7A). The same result was also obtained from sperm which was cocultured with oocyte for 1 h in mTBM medium. Culturing of IVF embryos in PZM-3 medium for 18 h resulted in the formation of two pronuclei. H3K36me3 was present at levels that were very low or undetectable by immunostaining in the two pronuclei (Fig. 7A). However, injection of a single fetal fibroblast cell into the perivitelline space of a denucleated oocyte without activation (Embedded) led to the development of a strong H3K36me3 signal in the cell (Fig. 7B). Activation of the restructured embryo with an electric pulse followed by culturing for 1 to 18 h in PZM-3 medium resulted in a gradual decrease in the H3K36me3 signal; however, H3K36me3 could still be clearly detected in the pronucleus. H3K36me3 steadily decreased until the 2-cell stage and then increased at the 4-cell stage; however, the intensities were not significantly different among 1-cell, 2-cell and 4-cell stage (Fig. 7C). From beyond the 8-cell stage to the blastocyst stage, H3K36me3 could not be detected in the embryos.

## Discussion

Histone H3K36 methylation has been extensively studied in yeast [25] and Drosophila [38], [39], where it is associated with transcription elongation. In yeast, H3K36 methylation is mediated by Set2 (histone lysine methyltransferase), which has been found to be associated with the elongation-competent form of RNA polymerase II [40], [41]. Transcription is a three-stage process consisting of initiation, elongation and termination; each stage requires a specific set of regulatory factors [42], [43]. During the elongation process, Set2, which has been found to catalyze the transfer of multiple methyl groups onto the H3K36 residue, is recruited to RNA polymerase II. This suggests that H3K36 methylation has a role in transcription elongation [24]. Morris et al. have demonstrated that H3K36 methylation is associated with the transcription-elongation process, showing that this modification is highly enriched over the transcribed regions of several active genes in *Schizosaccharomyces pombe* [24]. However, the association of histone H3K36 methylation with transcriptional activity in porcine somatic cells has not been reported.

In the current study, we report the first investigation of histone H3K36 methylation status and its association with transcriptional activity in porcine fetal fibroblasts. Using an immunocytochemical approach, we detected all three types of H3K36 methylations (me1, me2, and me3) in interphase cells. In a recent study, Li et al. also reported formation of H3K36me3 foci in interphase human HeLa cells [44]. This report and our data collectively suggest that this epigenetic modification (H3K36me3) may be conserved between pigs and humans. Using incorporation of FU into nascent RNA to identify transcriptionally active sites, dot-like structures were seen in nucleoplasm (Fig. 1), which are specific to Pol II activity [45]. Unlike H3K36me1 and -me2, which were distributed uniformly throughout the nucleoplasm, H3K36me3 was distributed within the nucleoplasm as discrete foci, a pattern similar to that displayed by transcriptionally active sites. This similar distribution pattern is consistent with an association of H3K36me3 with transcription. We also provided more direct evidence for this association by analyzing transcriptionally active sites containing FU-labeled nascent RNA. This analysis showed that only H3K36me3 strictly colocalized with transcriptionally active sites (Fig. 1), suggesting that H3K36me3, but not H3K36me1 or -me2, may be associated with transcription in porcine somatic cells. Luo et al. reported that H3K36me3 colocalized with POLII-Ser2-*P* which is the elongation form of RNA polymerase II in the mouse embryonic stem cells [46]. This report also suggests H3K36me3 associates with transcription elongation in mouse embryonic stem cell; this result is similar with our data. To further document the association of H3K36me3 with transcription, we treated porcine fetal fibroblasts with flavopiridol, which inhibits RNA polymerase II-mediated transcription in vitro and in vivo by blocking the transition to productive elongation [36], [37]. In the presence of flavopiridol, transcriptional activity was inhibited in interphase cells, as evidenced by a lack of FU signal which labeled active transcription sites (Fig. 2). Notably, H3K36me3 signals were also absent under these conditions, indicating an association of H3K36me3 with transcription elongation. In contrast, nuclear H3K36me1 and -me2 signals still remained, indicating that H3K36me1 and -me2 were not influenced by transcription elongation.

We also examined changes in H3K36 methylation status in porcine oocytes, preimplantation parthenogenetic activation embryos, and IVF embryos. Both H3K36me1 and -me2 were present in GV, MI and MII oocytes, and in all preimplantation stages of parthenogenetically activated embryos. The status of these two types of modification did not change during embryo preimplantation development. This may be because these two modifications have no function in regulating transcription following genome activation in the embryo. H3K36me3 status exhibited a different pattern. Although H3K36me3 was present in GV oocytes, its fluorescence intensity was low. However, H3K36me3 signals subsequently increased, becoming strong in MI and MII stage oocytes (Fig. 5). In these latter oocytes, the nucleus contained a more condensed chromosome compared with the chromatin in the GV stage oocyte. MI and MII stage oocytes also lack a nuclear membrane, allowing the antibody to more easily access to the chromosome. This may be why MI and MII stage oocytes exhibited a stronger H3K36me3 status than GV stage oocytes (Fig. 5). In matured oocytes activated by an electric pulse or IVF, H3K36me3 decreased and in some cases disappeared, suggesting the operation of an as yet unknown demethylation mechanism in the nuclei of oocytes during this process. Overexpression of JMJD2A, a histone demethylase of the JMJD2 family, has been shown to reverse tri-methylated H3K9 or H3K36 to di-methylated or unmethylated products in cultured human HeLa cells [47]. In contrast, RNA interference (RNAi)-mediated depletion of the JMJD2A homolog of *Caenorhabditis elegans* resulted in an increase in H3K9/K36 tri-methylation level in meiotic chromosomes. JMJD2C, another member of the JMJD2 demethylase family, also exhibits demethylation activity toward H3K36me3. Thus, it is possible that the demethylation of H3K36me3 observed in 1-cell stage embryos might be mediated by the JMJD2A or JMJD2C demethylation pathway; although further studies will be required to definitively establishing this.

Embryonic gene activation (EGA) is the process by which an embryo begins to transcribe its newly formed genome. EGA is essential for the synthesis of new proteins and subsequent cleavage events. It has been reported that porcine EGA occurs during the 4-cell stage [48], [7]. In the current study, H3K36me3 signals were present in both parthenogenetically activated and IVF 2-cell stage embryos. Although the fluorescence intensity was not strong, it was nonetheless detectable. The H3K36me3 modification continuously accumulated, reaching its highest level in 4-cell stage embryos (Figs. 5 and 6). These results suggest that tri-methylation of H3K36 starts at the 2-cell stage and enriched at the 4-cell stage, which corresponds well with the time of EGA in the pig. In the mouse, the major embryonic gene activation occurs in 2-cell stage; however, there is a minor embryonic gene activation in the 1-cell stage which has small quantity of transcription [49]. Similarly, tri-methylation of H3K36 starts in porcine 2-cell stage embryos may be related with the minor embryonic gene activation by the observation that small quantities of transcripts were produced in the 2-cell stage (our unpublished data). During the EGA process, the porcine 4-cell stage embryo starts transcribing its newly formed genome. Accordingly, these results suggest that H3K36me3 might be related to EGA and transcription during the preimplantation development of the porcine embryo. This result is similar to the recent study that showed the emergence of H3K36me3 at the time of zygotic-to-embryonic transition in zebra fish [50]. During transcription elongation in yeast, Set2-mediated H3K36 methylation coordinates the recruitment and activation of the Rpd3S complex over the coding region resulting in the maintenance of a hypoacetylated state and the repression of cryptic transcription [42]. H3K36 methylation affects transcription elongation via this Set2/Rpd3S pathway. In humans and mice, H3K36 tri-methylation is regulated by SETD2 methyltransferase (also known as KMT3A/SET2 or HYPB), which acts as the ortholog of yeast Set2 [51], [52], [53]. Human H3K36 methylation is mediated by SETD2, which associates with hyperphosphorylated RNA polymerase II and may play an important role in transcriptional regulation [54]. In our study, tri-methylation of H3K36 began in the 2-cell stage embryo; SETD2, or a similar methyltransferase, may be gradually recruited to RNA polymerase II and thereby regulate H3K36me3 modifications at that stage which may contain minor EGA. Subsequently, tri-methylation of H3K36 mediated by SETD2 (or a similar methyltransferase) peaked in 4-cell stage embryos and major EGA started. Additional studies will be required to establish the identity of the methyltransferase that coordinates porcine H3K36 tri-methylation and the regulatory mechanisms underlying it.

After the 8-cell stage and continuing to the blastocyst stage, H3K36me3 was no longer present in embryos. This may be because, after genome activation and formation of the new embryonic genome, H3K36me3 might not be necessary for transcription after the 8-cell stage. Alternatively, some other mechanism may release the tri-methylation of H3K36 and recruit other factors that regulate the transcription and development of the embryo. It has been shown that H3K36me3 is related to paused polymerase and Super Elongation Complex especially in developmentally regulated genes [55]. One can speculate that the rise of H3K36me3 at 4-cell stage is a response to an extensive polymerase pause release that promotes EGA and that does not happen again until blastocyst. However, Bošković et al. reported that, in mouse, H3K36me3 was only present in maternal chromatin immediately after fertilization and was absent in other stages of embryos, but was absent in all stages of bovine preimplantation embryos after fertilization [56]. These observations contrast with our results, possibly because of differences in the antibodies used. In preliminary experiments, we tested different H3K36me3 antibodies from different vendors and were able to obtain a signal using only the one reported here. We also increased the intensity of the permeabilization step of the immunofluorescence staining protocol, which could have improved the ability of the antibody to access embryonic nuclei.

An important factor underlying the low efficiency of SCNT is the incomplete reprogramming of donor cell nuclei. In SCNT, the nucleus of the donor cell carries its own epigenetic structures, which are different from those of gametes [57]. After nuclear transfer, abnormal epigenetic modifications occur and induce incomplete reprogramming of the donor nucleus. For example, the acetylation of H4K8 and H4K12 in the somatic nucleus after SCNT is incomplete [58], and H3K27me3 signals in blastocysts produced by SCNT are different from those of IVF blastocysts [59]. Our data showed that H3K36me3 is present in porcine fetal fibroblasts (Fig. 1), but is present at a very low level, or is even absent, in sperm (Fig. 7A). This difference in H3K36me3 status between somatic cells and sperm may lead to aberrant histone methylation in the SCNT embryo. Upon injection of donor porcine fetal fibroblasts into the perivitelline space of denucleated oocytes without electric pulse activation, a strong H3K36me3 signal was observed (Fig. 7B). After activation and culture for 1 h, H3K36me3 was still present in the restructured embryo. After culturing for 18 h, a pronucleus formed in the restructured embryo. H3K36me3 decreased to some extent; however, it still could be clearly detected. During this stage, IVF embryos underwent demethylation of H3K36me3 (Fig. 7A), whereas aberrant tri-methylation of H3K36 occurred in SCNT embryos. H3K36me3 could not be demethylated completely during the early 1-cell stage in SCNT embryos. Interestingly, the H3K36me3 signal became weak at the 2-cell stage, indicating that the nucleus of the restructured embryo was gradually demethylated by the denucleated cytoplasm prior to this point. However, there were no significant differences of the intensities of H3K36me3 among 1-cell, 2-cell and 4-cell stage NT embryos (Fig. 7C); this result indicates the demethylation of H3K36me3 in 1-cell stage of NT embryo could not be sufficient compared with IVF or parthenogenetic embryos. When the embryo reached the 4-cell stage, H3K36me3 signals increased again and then decreased after the 8-cell stage, indicating demethylation. The changes in H3K36me3 status between the 4-cell and blastocyst stages were similar between SCNT and IVF embryo.

Collectively, our data indicate that H3K36me3 is associated with the elongation phase of transcription in porcine fetal fibroblasts. Developmentally regulated H3K36me3, but not -me1 or -me2, may be a histone mark of EGA in the pig. Aberrant H3K36 tri-methylation occurs during the 1-cell stage in porcine SCNT embryos. The mechanisms underlying the interaction of H3K36me3 with transcription elongation and the effects of abnormal H3K36 tri-methylation on the development of SCNT embryos require further investigation.

## References

[1] Worrad DM, Ram PT, Schultz RM (1994) Regulation of gene expression in the mouse oocyte and early preimpantation embryo: developmental changes in Sp1 and TATA box-binding protein, TBP. Development 120: 2347–2357. 7925035 10.1242/dev.120.8.2347

[2] Latham KE, Schultz RM (2001) Embryonic genome activation. Front Biosci 6: 748–759. 10.2741/latham 11401780

[3] Kanka J (2003) Gene expression and chromatin structure in the pre-implantation embryo. Theriogenology 59: 3–19. 12499014 10.1016/s0093-691x(02)01267-0

[4] Hamatani T, Carter MG, Sharov AA, Ko MS (2004) Dynamics of global gene expression changes during mouse preimplantation development. Dev Cell 6: 117–131. 14723852 10.1016/s1534-5807(03)00373-3

[5] Wang QT, Piotrowska K, Ciemerych MA, Milenkovic L, Scott MP, et al (2004) A genome-wide study of gene activity reveals developmental signaling pathways in the preimplantation mouse embryo. Dev Cell 6: 133–44. 14723853 10.1016/s1534-5807(03)00404-0

[6] Schultz M (1993) Regulation of zygotic gene activation in the mouse. Bioessays 15: 531–538. 8135766 10.1002/bies.950150806

[7] Bjerregaard B, Pedersen HG, Jakobsen AS, Rickords LF, Lai L, et al (2007) Activation of ribosomal RNA genes in porcine embryos produced in vitro or by somatic cell nuclear transfer. Mol Reprod Dev 74: 35–41. 16941707 10.1002/mrd.20594

[8] Barnes FL, First NL (1991) Embryonic transcription in vitro cultured bovine embryos. Mol Reprod Dev 29: 117–123. 1878221 10.1002/mrd.1080290205

[9] Mason K, Liu Z, Aguirre-Lavin T, Beaujean N (2012) Chromatin and epigenetic modifications during early mammalian development. Anim Reprod Sci 134: 45–55. 22921722 10.1016/j.anireprosci.2012.08.010

[10] Dillon N, Festenstein R (2002) Unravelling heterochromatin: competition between positive and negative factors regulates accessibility. Trends Genet 18: 252–258. 12047950 10.1016/s0168-9525(02)02648-3

[11] Kouzarides T (2007) Chromatin modifications and their function. Cell 128: 693–705. 17320507 10.1016/j.cell.2007.02.005

[12] Li B, Carey M, Workman JL (2007) The role of chromatin during transcription. Cell 128: 707–719. 17320508 10.1016/j.cell.2007.01.015

[13] Zhang Y, Reinberg D (2001) Transcription regulation by histone methylation: interplay between different covalent modifications of the core histone tails. Genes Dev 15: 2343–2360. 11562345 10.1101/gad.927301

[14] Martin C, Zhang Y (2005) The diverse functions of histone lysine methylation. Nat Rev Mol Cell Biol 6: 838–849. 16261189 10.1038/nrm1761

[15] Ng HH, Robert F, Young RA, Struhl K (2003) Targeted recruitment of Set1 histone methylase by elongating Pol II provides a localized mark and memory of recent transcriptional activity. Mol Cell 11: 709–719. 12667453 10.1016/s1097-2765(03)00092-3

[16] Surani MA, Hayashi K, Hajkova P (2007) Genetic and epigenetic regulators of pluripotency. Cell 128: 747–762. 17320511 10.1016/j.cell.2007.02.010

[17] Santos F, Peters AH, Otte AP, Reik W, Dean W (2005) Dynamic chromatin modifications characterise the first cell cycle in mouse embryos. Dev Biol 280: 225–236. 15766761 10.1016/j.ydbio.2005.01.025

[18] Schneider R, Bannister AJ, Myers FA, Thorne AW, Crane-Robinson C, et al (2004) Histone H3 lysine 4 methylation patterns in higher eukaryotic genes. Nat Cell Biol 6: 73–77. 14661024 10.1038/ncb1076

[19] Schübeler D, MacAlpine DM, Scalzo D, Wirbelauer C, Kooperberg C, et al (2004) The histone modification pattern of active genes revealed through genome-wide chromatin analysis of a higher eukaryote. Genes Dev 18: 1263–1271. 15175259 10.1101/gad.1198204 PMC420352

[20] Miao F, Natarajan R (2005) Mapping global histone methylation patterns in the coding regions of human genes. Mol Cell Biol 25: 4650–4661. 15899867 10.1128/MCB.25.11.4650-4661.2005 PMC1140629

[21] Zhao XD, Han X, Chew JL, Liu J, Chiu KP, et al (2007) Whole-genome mapping of histone H3 Lys4 and 27 trimethylations reveals distinct genomic compartments in human embryonic stem cells. Cell Stem Cell 1: 286–298. 18371363 10.1016/j.stem.2007.08.004

[22] Sims RJ III, Nishioka K, Reinberg D (2003) Histone lysine methylation: a signature for chromatin function. Trends Genet 19: 629–639. 14585615 10.1016/j.tig.2003.09.007

[23] Pan G, Tian S, Nie J, Yang C, Ruotti V, et al (2007) Whole-genome analysis of histone H3 lysine 4 and lysine 27 methylation in human embryonic stem cells. Cell Stem Cell 1: 299–312. 18371364 10.1016/j.stem.2007.08.003

[24] Morris SA, Shibata Y, Noma K, Tsukamoto Y, Warren E, et al (2005) Histone H3 K36 methylation is associated with transcription elongation in Schizosaccharomyces pombe, Eukaryot Cell. 4: 1446–1454. 10.1128/EC.4.8.1446-1454.2005 PMC1214526 16087749

[25] Pokholok DK, Harbison CT, Levine S, Cole M, Hannett NM, et al (2005) Genome-wide map of nucleosome acetylation and methylation in yeast. Cell 122: 517–527. 16122420 10.1016/j.cell.2005.06.026

[26] Rao B, Shibata Y, Strahl BD, Lieb JD (2005) Dimethylation of histone H3 at lysine 36 demarcates regulatory and nonregulatory chromatin genome-wide. Mol Cell boil 25: 9447–9459. 10.1128/MCB.25.21.9447-9459.2005 PMC1265832 16227595

[27] Barski A, Cuddapah S, Cui K, Roh TY, Schones DE, et al (2007) High-resolution profiling of histone methylation in the human genome. Cell 129: 823–837. 17512414 10.1016/j.cell.2007.05.009

[28] Joshi AA, Struhl K (2005) Eaf3 chromodomain interaction with methylated H3–K36 links histone deacetylation to Pol II elongation. Mol Cell 20: 971–978. 16364921 10.1016/j.molcel.2005.11.021

[29] Keogh MC, Kurdistani SK, Morris SA, Ahn SH, Podolny V, et al (2005) Cotranscriptional set2 methylation of histone H3 lysine 36 recruits a repressive Rpd3 complex. Cell 123: 593–605. 16286008 10.1016/j.cell.2005.10.025

[30] Lee JS, Shilatifard A (2007) A site to remember: H3K36 methylation a mark for histone deacetylation. Mutation Research 618: 130–134. 17346757 10.1016/j.mrfmmm.2006.08.014

[31] Carrozza MJ, Li B, Florens L, Suganuma T, Swanson SK, et al (2005) Histone H3 methylation by Set2 directs deacetylation of coding regions by Rpd3S to suppress spurious intragenic transcription. Cell 123: 581–592. 16286007 10.1016/j.cell.2005.10.023

[32] Li B, Gogol M, Carey M, Pattenden SG, Seidel C, et al (2007) Infrequently transcribed long genes depend on the Set2/Rpd3S pathway for accurate transcription. Genes Dev 21: 1422–1430. 17545470 10.1101/gad.1539307 PMC1877753

[33] Venkatesh S, Smolle M, Li H, Gogol MM, Saint M, et al (2012) Set2 methylation of histone H3 lysine 36 suppresses histone exchange on transcribed genes. Nature 489: 52–455. 22914091 10.1038/nature11326

[34] Li XX, Lee DS, Kim KJ, Lee JH, Kim EY, et al (2013) Leptin and nonessential amino acids enhance porcine preimplantation embryo development in vitro by intracytoplasmic sperm injection. Theriogenology 79: 291–298. 23174770 10.1016/j.theriogenology.2012.08.019

[35] Diao YF, Naruse KJ, Han RX, Li XX, Oqani RK, et al (2013) Treatment of fetal fibroblasts with DNA methylation inhibitors and/or histone deacetylase inhibitors improves the development of porcine nuclear transfer-derived embryos. Anim Reprod Sci 141: 164–171. 24021942 10.1016/j.anireprosci.2013.08.008

[36] Chao SH, Fujinaga K, Marion JE, Taube R, Sausville EA, et al (2000) Flavopiridol inhibits P-TEFb and blocks HIV-1 replication. J Biol Chem 275: 28345–28348. 10906320 10.1074/jbc.C000446200

[37] Chao SH, Price DH (2001) Flavopiridol inactivates P-TEFb and blocks most RNA polymerase II transcription in vivo. J Biol Chem 276: 31793–31799. 11431468 10.1074/jbc.M102306200

[38] Stabell M, Larsson J, Aalen RB, Lambertsson A (2007) Drosophila dSet2 functions in H3-K36 methylation and is required for development. Biochem Biophys Res Commun 359: 784–789. 17560546 10.1016/j.bbrc.2007.05.189

[39] Bell O, Wirbelauer C, Hild M, Scharf AN, Schwaiger M, et al (2007) Localized H3K36 methylation states define histone H4K16 acetylation during transcriptional elongation in Drosophila. EMBO J 26: 4974–4984. 18007591 10.1038/sj.emboj.7601926 PMC2140113

[40] Gerber M, Shilatifard A (2003) Transcriptional elongation by RNA polymerase II and histone methylation. J Biol Chem 278: 26303–26306. 12764140 10.1074/jbc.R300014200

[41] Krogan NJ, Kim M, Tong A, Golshani A, Cagney G, et al (2003) Methylation of histone H3 by Set2 in Saccharomyces cerevisiae is linked to transcriptional elongation by RNA polymerase II. Mol Cell Biol 23: 4207–4218. 12773564 10.1128/MCB.23.12.4207-4218.2003 PMC427527

[42] Venkatesh S, Workman JL (2013) Set2 mediated H3 lysine 36 methylation: regulation of transcription elongation and implications in organismal development. WIREs Dev Biol doi: 10.1002/wdev.109. 10.1002/wdev.109 PMC3767924 24014454

[43] Sims RJ 3rd, Belotserkovskaya R, Reinberg D (2004) Elongation by RNA polymerase II: the short and long of it. Genes Dev 18: 2437–2468. 15489290 10.1101/gad.1235904

[44] Li F, Mao GG, Tong D, Huang J, Gu LY, et al (2013) The Histone Mark H3K36me3 Regulates Human DNA Mismatch Repair through Its Interaction with MutSα. Cell 153: 590–600. 23622243 10.1016/j.cell.2013.03.025 PMC3641580

[45] Xie SQ, Martin S, Guillot PV, Bentley DL, Pombo A (2006) Splicing speckles are not reservoirs of RNA polymerase II, but contain an inactive form, phosphorylated on serine2 residues of the C-terminal domain. Mol Biol Cell 17: 1723–1733. 16467386 10.1091/mbc.E05-08-0726 PMC1415300

[46] Luo L, Gassman KL, Petell LM, Wilson CL, Bewersdorf J, et al (2009) The nuclear periphery of embryonic stem cells is a transcriptionally permissive and repressive compartment. J Cell Sci 122: 3729–3737. 19773359 10.1242/jcs.052555 PMC2758804

[47] Whetstine JR, Nottke A, Lan F, Huarte M, Smolikov S, et al (2006) Reversal of Histone Lysine Trimethylation by the JMJD2 Family of Histone Demethylases. Cell 125: 467–481. 16603238 10.1016/j.cell.2006.03.028

[48] Hyttel P, Laurincik J, Rosenkranz Ch, Rath D, Niemann H, et al (2000) Nucleolar proteins and ultrastructure in preimplantation porcine embryos developed in vivo. Biol Reprod 63: 1848–1856. 11090457 10.1095/biolreprod63.6.1848

[49] Bellier S, Chastant S, Adenot P, Vincent M, Renard JP, et al (1997) Nuclear translocation and carboxyl-terminal domain phosphorylation of RNA polymerase II delineate the two phases of zygotic gene activation in mammalian embryos. EMBO J 16: 6250–6262. 9321404 10.1093/emboj/16.20.6250 PMC1326309

[50] Vastenhouw NL, Zhang Y, Woods IG, Imam F, Regev A, et al (2010) Chromatin signature of embryonic pluripotency is established during genome activation. Nature 464: 922–926. 20336069 10.1038/nature08866 PMC2874748

[51] Yuan W, Xie J, Long C, Erdjument-Bromage H, Ding X, et al (2009) Heterogeneous nuclear ribonucleoprotein L is a subunit of human KMT3a/Set2 complex required for H3 Lys-36 trimethylation activity in vivo. J Biol Chem 284: 15701–15707. 19332550 10.1074/jbc.M808431200 PMC2708867

[52] Hu M, Sun XJ, Zhang YL, Kuang Y, Hu CQ, et al (2010) Histone H3 lysine 36 methyltransferase Hypb/Setd2 is required for embryonic vascular remodeling. Proc Natl Acad Sci USA 107: 2956–2961. 20133625 10.1073/pnas.0915033107 PMC2840328

[53] Edmunds JW, Mahadevan LC, Clayton AL (2008) Dynamic histone H3 methylation during gene induction: HYPB/Setd2 mediates all H3K36 trimethylation. EMBO J 27: 406–420. 18157086 10.1038/sj.emboj.7601967 PMC2168397

[54] Sun XJ, Wei J, Wu XY, Hu M, Wang L, et al (2005) Identification and Characterization of a Novel Human Histone H3 Lysine 36-specific Methyltransferase. J Biol Chem 280: 35261–35271. 16118227 10.1074/jbc.M504012200

[55] Lin C, Garrett AS, De Kumar B, Smith ER, Gogol M, et al (2011) Dynamic transcriptional events in embryonic stem cells mediated by the super elongation complex (SEC). Gene Dev 25: 1486–1498. 21764852 10.1101/gad.2059211 PMC3143939

[56] Bošković A, Bender A, Gall L, Ziegler-Birling C, Beaujean N, et al (2012) Analysis of active chromatin modifications in early mammalian embryos reveals uncoupling of H2A.Z acetylation and H3K36 trimethylation from embryonic genome activation. Epigenetics 7: 747–757. 22647320 10.4161/epi.20584

[57] Breton A, LE Bourhis D, Audouard C, Vignon X, Lelièvre JM (2010) Nuclear profiles of H3 histones trimethylated on Lys27 in bovine (Bos taurus) embryos obtained after in vitro fertilization or somatic cell nuclear transfer. J Reprod Dev 56: 379–388. 20431250 10.1262/jrd.09-182a

[58] Wang F, Kou Z, Zhang Y, Gao S (2007) Dynamic reprogramming of histone acetylation and methylation in the first cell cycle of cloned mouse embryos. Biol Reprod 77: 1007–1016. 17823087 10.1095/biolreprod.107.063149

[59] Zhang M, Wang F, Kou Z, Zhang Y, Gao S (2009) Defective chromatin structure in somatic cell cloned mouse embryos. J Biol Chem 284: 24981–24987. 19602512 10.1074/jbc.M109.011973 PMC2757202

